# Aging and Mixed Emotions: A Word-Suffix Approach in Free Recall

**DOI:** 10.3390/bs13020160

**Published:** 2023-02-12

**Authors:** Rocco Palumbo, Alberto Di Domenico, Nicola Mammarella

**Affiliations:** Department of Psychological Sciences, Health and Territory, University G. d’Annunzio of Chieti-Pescara, 66100 Chieti, Italy

**Keywords:** aging, recall, emotions, cognition, memory

## Abstract

The current study investigated mixed-emotional memories in groups of young, young-old, and old-old participants. We used a “word-suffix approach” to simulate the co-occurrence of positive and negative emotions. The participants engaged in a free-recall task for valenced words and mixed-emotional words (valenced words coupled with pejorative or endearment suffixes). Our results showed that the groups of older adults recalled higher numbers of suffixed words compared to their younger counterparts. Our findings highlighted older adults’ tendency to perceive and remember emotionally ambivalent words to a greater extent than younger adults and showed that the young-old participants were particularly good at solving ambivalence by focusing on positive-dominant ambivalent words.

## 1. Introduction

When positive and negative emotions co-occur, people may experience mixed emotions [1,2]. Mixed emotions refer to conditions that have both pleasant and unpleasant aspects (i.e., feeling happy and sad at the same time). Mixed emotions have been shown to arise especially in situations when people experience emotional ambivalence (e.g., while watching a movie or listening to a song [3,4]). Although research on mixed emotions varies in terms of methodology and theoretical framework [5], there is a large consensus that two emotions with opposite valences can co-exist. In the aging literature, the term “mixed emotions” has often been used interchangeably with the conceptualization of poignancy. This approach was mainly adopted to better highlight the temporal dimension that may characterize mixed feelings. 

In particular, Carstensen and colleagues [6] pointed to the fact that poignancy is higher when people are reminded of time constraints and perceive that time is ending. In line with this idea that time constraints enhance mixed feelings, previous research has demonstrated that older adults experience poignancy more frequently than younger adults [6,7,8,9,10]. 

These results have been explained in terms of the socio-emotional selectivity theory as follows [11]. First, older adults perceive the end of life as being nearer than their younger counterparts and thus place greater emphasis on emotionally complex goals. Second, older adults are more likely than younger adults to experience ambivalent situations due to their age and adult responsibilities (e.g., they are more likely to become a grandfather or grandmother or to experience the death of a partner or close friend). These conditions increase older adults’ awareness that each moment may be their last and thus give rise to rich and complex emotional reactions. 

Third, the direction of valenced biases, typically shown during cognitive tasks, reflects older adults’ emotion-regulation goals. In particular, healthy older adults focus on positively charged information to a greater extent than younger adults when facing ambivalent situations [12,13,14,15]. Focusing on positively charged information allows older adults to achieve emotional well-being in the time they have left. This was shown, for instance, by Bucks et al. [16], who found that older adults were significantly less likely than younger adults to report the presence of anger in angry–happy face mixes.

More recent studies on mixed emotions have identified multiple factors that are involved in the generation of mixed emotions, especially in the context of aging research. To cite only a few, these factors include the effects of contextual factors and individual differences [17], levels of cognitive functioning [18,19], and emotion-regulation goals [20,21].

In this regard, another interesting approach is the one put forward by Harmon-Jones and colleagues [22]. They claim that the accessibility in memory of a positive attitude toward a domain might increase one’s motivation toward that domain. Positivity effects in memory in older adults may thus derive from their positive attitudes and motivation to remember a greater number of positively laden words. This attitude-based approach may be considered complimentary to that of Ersner-Hershfield and colleagues [11], as it assumes that motivational goals drive older adults’ focus on emotionally ambivalent words to a greater extent than in younger adults.

Many studies have used the manipulation of hybrid faces, i.e., positive and negative emotions mixed to different degrees in a face (e.g., an anger expression mixed with a happy expression), to elicit mixed emotions, as shown by Bucks and colleagues [16]. Other studies have used sentences, stories, or word pairs [23,24,25,26,27]. More specifically, in the verbal domain, a series of studies by Briesemeister and colleagues [28,29] showed, for instance, that when rated separately for positivity and negativity, some words score high on both scales. Thus, words, too, can be perceived as being positive and negative at the same time. A typical example of an ambivalent word is “school”. Other studies [30] have also pointed to the bivalent nature of words in other languages, such as Spanish.

In this study, to test whether mixed emotions can be elicited by words, we used a slightly different approach, which we called a “word-suffix approach”. Unlike English, Italian grammar is characterized by a higher number of suffixes that may be linked to a word and consequently change or modulate its emotional meaning. In particular, we used pejorative and endearment suffixes to elicit mixed emotions. A suffix is said to be pejorative when it attaches a negative meaning to a word. Examples of English pejorative suffixes are -aster or -ster (e.g., poetaster, oldster) and -ard (e.g., sluggard). In Italian, pejorative suffixes are numerous, e.g., -accio and -aglia (ragazzaccio = bad boy, gentaglia = bad people), -ume and -ucolo (e.g., politicume = bad politics, avvocatucolo = inexperienced and cheap lawyer), etc. By contrast, an endearment suffix (“vezzeggiativo” in Italian) attaches a positive meaning to a word, e.g., the suffixes -y in doggy and -let in droplet, in English, and the suffixes -uccio, -ino, and -etto (e.g., sonaglino, orsetto), in Italian. In order to develop stimuli with mixed emotional valences, we attached a pejorative suffix to a series of positive words and an endearment suffix to a series of negative words. 

Carstensen et al. [7] showed that poignancy is increased in older adults and that it is therefore reflected in their memory performance. Consequently, generally speaking, we expected older adults to focus on and remember a higher number of mixed-emotional words compared to younger adults. We assessed this hypothesis by asking groups of young, young-old, and old-old adults to engage in a free-recall task for suffixed words among positive, negative, and neutral words. In this way, we investigated whether memory for mixed emotions changes over time.

Differently from Briesemeister and colleagues [28,29], our suffix approach allows us to test another interesting assumption, namely, that a greater focus on positive information helps to solve ambivalence. We compared memory for positive words that were coupled with a pejorative suffix and memory for negative words that were coupled with an endearment suffix. We reasoned that, if older adults tend to focus on positive information to solve emotional ambivalence, we should observe better memory for positive words coupled with a pejorative suffix compared to negative words coupled with an endearment suffix. That is, older adults should focus on and remember a higher number of words that have a dominant positive connotation (e.g., amoraccio = bad love) compared to words that have a dominant negative connotation (e.g., disastruccio = little disaster). In addition, we expected age-related differences. Positive and negative mixed words were expected to be recalled to a similar extent in young individuals, whereas, generally speaking, older adults were expected to recall more positive mixed words than negative mixed words. More specifically, in terms of differences between the young-old and the old-old groups, we were able to observe an interesting pattern. In fact, it is possible that the predominance of positive-dominant ambivalent words occurs in the young-old group solely due to a typically positive focus in their cognitive processing. By contrast, the old-old group may show no differences in terms of positive- vs. negative-dominant ambivalent words in their recall. On the one hand, this may be due to their reduced cognitive abilities and controlled processes that do not favor predominantly positive information processing [31]. On the other hand, studies [32] have shown that, as older adults (e.g., those close to 100 years old) approach the end of their lives, they use a more neutral or detached type of emotional processing as a regulation strategy which is typically reflected in their recall in terms of similar numbers of positive and negative words or mixed patterns of data.

## 2. Materials and Methods

### 2.1. Participants

Twenty-four young (50% female; aged 18–25, mean = 21.92, SD = 2.55), 24 young-old (50% female; aged 65–73, mean = 68.62, SD = 2.70), and 24 old-old adults (50% female; aged 75–85, mean = 79.42, SD = 2.92) participated in the study. The younger adults were undergraduates at the University of Chieti who participated voluntarily in exchange for psychology course credit. Older adults did not receive monetary reimbursement for participation, and some of them took part in other experimental sessions in our lab. Exclusion criteria included treatment for memory problems, head injuries resulting in hospitalization for more than 24 h, and/or medical conditions that could potentially affect cognitive functioning (e.g., Alzheimer’s disease, multiple sclerosis, and Parkinson’s disease). Additionally, all older participants reported being in good mental and physical health and without major hearing or vision problems. Before taking part in the experiment, the participants were administered a series of cognitive and affective screening tests. We used the classical forward and backward digit span [33] to evaluate working-memory capacity, the phonemic fluency test [33] to measure verbal fluency, and the Positive and Negative Affective Scale (PANAS) [34] to evaluate positive and negative feelings (means are reported in Table 1). Older adults were also administered the Mini Mental State Exam (MMSE; [35]) to measure general cognitive functioning and the Geriatric Depression Scale (GDS; [36]) to assess negative thoughts and depression. All the participants were healthy individuals since they did not meet the criteria for cognitive impairment or depression. Younger adults were screened for depression using the Beck Depression Inventory [37]. The participants were tested during an experimental window of 2 months, just before the second wave of the COVID-19 pandemic hit.

### 2.2. Design

The study design was a 3 (age: young, young-old, old-old) x 5 (word valence: positive, negative, neutral, mixed-positive words and mixed-negative words) mixed-factorial design, with age as the between-group variable and word valence as the repeated-measures factor. The dependent variable was the proportion of correct recall.

### 2.3. Material

A total of 216 words were pulled from the Italian adaptation of the ANEW [38]. From these words, we selected a total of 72 positive words, 72 negative words, and 36 neutral words. Only words with high usage frequencies were selected. Frequencies were determined based on the ColFIS database [39], which provides frequencies for both lemmas and forms of over 3 million words, reflecting the reading habits of the Italian population. Only words with lemmas above the median were selected. The words were selected to be different in valence but with similar levels of arousal, frequency, and imaginability. The set with the 72 positive words was further divided into two subsets of 36 items each: 36 were used as positively valenced words and 36 were used for the mixed-emotion manipulation. The same procedure was adopted for the set of 72 negative words. In particular, mixed-emotional words were formed by adding a pejorative (e.g., -aster) suffix to positive words (for a total of 36 suffixed positive words) or an endearment suffix (-y) to negative words (for a total of 36 suffixed negative words). These 72 suffixed words were previously rated by a group of 24 participants as highly ambivalent on a 9-point scale (from 1 = not at all to 9 = absolutely). The participants were asked to rate the degree of ambivalence assigned to each word and its corresponding valence and arousal. We found that positive words with a pejorative suffix were rated as highly ambivalent and more positively charged, while negative words with an endearment suffix were rated as highly ambivalent and more negative in valence. Altogether, the stimulus list was made up of 36 positive, 36 negative, and 36 neutral words, as well as 36 mixed-positive words (positive words with a pejorative suffix) and 36 mixed-negative words (negative words with an endearment suffix). Each list of 36 items was further divided into 9 subsets of 4 items each. In this way, we made up 9 lists of 20 items each mixed up in random orders (4 positive, 4 negative, 4 neutral, 4 positive with a pejorative suffix, and 4 negative with an endearment suffix). The attribution of items to each list was randomly repeated for each subject. We presented lists of 20 items to avoid fatigue effects for the old-old group and reduce the level of task difficulty. Pilot studies showed that increasing the number of words per list (to more than 20 words) disrupted old-old recall.

### 2.4. Procedure

After signing their informed consent, the participants viewed a series of 9 lists of 20 words each. The words were presented one at a time in random order, at a rate of 2 s per word. Each word presentation began with a black fixation cross on a white background in the center of the screen for 500 ms, immediately followed by a word. The participants knew that their memory would be tested. After the presentation of each list, the participants were given 3 min to write down the words they could recall from the previously presented list. 

## 3. Results

A mixed 3 (age) x 5 (type of valence) ANOVA (Table 2) of proportions of correct recall was performed and followed by post hoc comparisons using the Tukey HSD test. Gender was not included in the analysis because no significant differences were found (F_(1,66)_ = 0.401, *p* = 0.529, η^2^ = 0.006). The results showed a main effect of age (F_(2,69)_ = 40.346, *p* < 0.001, η^2^ = 0.539), as old-old participants recalled fewer words (M = 0.487, SD = 0.110) compared to young-old (M = 0.594, SD = 0.115; *p* < 0.001) and young participants (M = 0.611, SD = 0.133; *p* < 0.001). No recall differences were detected between the young-old and young groups (*p* = 0.382). The results showed a main effect of type of valence (F_(4,276)_ = 22.490, *p* < 0.001, η^2^ = 0.0.246), which was due to positive (M = 0.615, SD = 0.127), negative (M = 0.584, SD = 0.149), *p* < 0.001), mixed-positive (M = 0.593, SD = 0.142), and mixed-negative words (M = 0.575, SD = 0.104) being better recalled than neutral words (M = 0.473, SD = 0.101; ps < 0.001). Negative and mixed words did not differ (*p* = 0.979), but both were recalled more than neutral ones (ps < 0.001).

The two-way interaction was significant (F_(8,276)_= 20.576, *p*< 0.001, η^2^ = 0.374). In the young group, negative words (M = 0.729, SD = 0.132) were recalled better than neutral (M = 0.553, SD = 0.062; *p* < 0.001), mixed-positive (M = 0.493, SD = 0.125; *p* < 0.001), mixed-negative (M = 0.516, SD = 0.112; *p* < 0.001), and positive words (M = 0.657, SD = 0.112; *p* < 0.01). Recall did not differ between neutral (M = 0.493, SD = 0.125), mixed-positive (M = 0.493, SD = 0.125; *p* = 0.730), and mixed-negative words (M = 0.516, SD = 0.112; *p* = 0.994). Positive words (M = 0.657, SD = 0.112) were recalled better than neutral (M = 0.553, SD = 0.062; *p* < 0.05), mixed-positive (M = 0.493, SD = 0.125), and mixed-negative words (M = 0.516, SD = 0.112; ps < 0.001). Although the young-old group recalled a similar number of positive (M = 0.675, SD = 0.118), mixed-positive (M = 0.714, SD = 0.119; *p* = 0.989), and mixed-negative words (M = 0.618, SD = 0.088; *p* = 0.807), they recalled more mixed-positive and mixed-negative words than negative (M = 0.543, SD = 0.077; ps < 0.001) and neutral ones (M = 0.493, SD = 0.063; ps < 0.001). Negative and neutral words were recalled to similar extents (*p* = 0.920).

Furthermore, in the old-old group, there was a higher number of mixed-positive (M = 0.572, SD = 0.081) and mixed-negative words (M = 0.591, SD = 0.084) recalled compared to positive (M = 0.512, SD = 0.083; *p* < 0.01), negative (M = 0.481, SD = 0.102; *p* < 0.001), and neutral words (M = 0.372, SD = 0.075; *p* < 0.001). Positive and negative word recall was similar (*p* = 0.999), and both sets of words were recalled more compared to neutral ones (ps < 0.001).

A subsequent analysis was performed on mixed words alone to see whether the participants recalled different numbers of types of mixed words. Specifically, we wanted to determine whether the participants recalled more positive-dominant words (positive words attached to a pejorative suffix) than negative-dominant words (negative words attached to an endearment suffix). These analyses showed a main effect of age (F_(2,69)_ = 29.516, *p* < 0.001, η^2^ = 0.461), as old-old participants (M = 0.582, SD = 0.082) recalled more mixed words than the young group (M = 0.505, SD = 0.118; *p* < 0.01), even though they recalled fewer mixed words than the young-old group (M = 0.666, SD = 0.115; *p* < 0.001). The young group recalled a lower number of words compared to the young-old (*p* < 0.001) and old-old groups (*p* < 0.01). The effect of type of mixed words was not significant (F_(1,69)_ = 1.063, *p* = 0.306, η^2^ = 0.015), as the participants recalled a similar number of positive-dominant words. 

Finally, the two-way interaction was significant (F_(2,69)_ = 5.177, *p* < 0.01, η^2^ = 0.130). The young and old-old groups recalled similar numbers of positive- (M = 0.493, SD = 0.125 and M = 0.572, SD = 0.081, respectively; *p* = 0.087) and negative-dominant words (M = 0.516, SD = 0.112 and M = 0.591, SD = 0.084, respectively; *p* = 0.116), but the young-old participants recalled more positive-dominant words (M = 0.714, SD = 0.119) compared to negative-dominant words (M = 0.618, SD = 0.088; *p* < 0.05). 

## 4. Discussion

This study used a word-suffix approach to assess mixed-emotional memories in aging. In line with previous studies that showed an increase in mixed emotions as we age [7,11], our study suggests that a greater focus on mixed emotions in aging leads older adults to remember greater numbers of mixed-emotional words. Furthermore, looking at mixed-emotional recall alone, we observed a bump in the young-old group, which may indicate that this age group can be more prone to ambivalent emotional situations compared to young-old and old-old groups. Consequently, their memories may become more sensitive to mixed-emotional stimuli. An interesting pattern of results was indeed found in this group. We replicated the classical positivity effect, as the participants recalled a higher number of positive words compared to negative words. In addition, the number of positive words recalled was similar to the number of mixed-emotional words recalled that were pooled from positive and negative words. When mixed-emotional words were further divided into words with a positive vs. negative dominant connotation, the participants showed a strong preference for positive words that were coupled with a pejorative suffix. This finding indicates that the young-old group’s cognitive resources are directed towards positively laden information to solve ambivalence. This pattern did not obtain for the old-old group. Although the participants showed a mixed-emotional enhancement effect, they were not able to prioritize positive over negative information in their recall. Altogether, our findings are in line with Mather and Knight’s model of cognitive control as an explanation of the positivity effect in the memory of older adults [40,41]. When older adults’ cognitive resources are able to prioritize positive information, they show a greater focus on positive information in their recall. However, when cognitive resources diminish, positivity biases may suffer. In addition, our data are also in line with studies showing a tendency among old-old individuals to focus less on emotional information due to their more detached type of emotional processing as a regulation strategy for the end of life. 

One main limitation of this study was the use of Italian mixed-emotional words in the experimental procedure, which prevented us from generalizing our results across different languages. Nevertheless, we believe that our manipulation helped capture emotional ambivalence effects in memory using only words as stimuli. Future studies may employ a similar word-suffix approach to simulate ambivalence in different languages and different cultures. In fact, recent studies have found that culture might influence the extent to which mixed emotions are perceived [26].

Furthermore, to provide more direct evidence of the allocation of attention towards the positive-dominant components of words in the young-old, future research could utilize more direct perceptual measures, based, for example, on online-elaboration and eye-movement data, which would allow the evaluation of changes in the distribution of processing resources towards mixed-emotional stimuli in general and positively laden information in particular. 

Some other limitations may be related to the fact that dispositional processes may have affected our results. For example, it has been shown that positive and negative affects may bias behavior toward approach to rewards and withdrawal from threat, particularly when the contingencies are ambiguous [42,43], indicating that mood may play an important role in valence attribution. In this regard, attitudes such as subjective evaluations that range from good to bad that are represented in memory may also influence emotional-situation selection [22,44]. It would be interesting in future studies to evaluate subjects’ attitudes towards positive and negative emotions and clarify whether attitudes affect the tendency in older adults to select, for instance, greater numbers of positive words with pejorative suffixes. Another concern may be related to the assumption that our results on aging could have been influenced by the participants’ perceptions regarding the unknown health impacts of the COVID-19 pandemic. In fact, we tested the participants right before the second wave of the pandemic hit. Consequently, the focus on ambivalence could be due to the lack of clear information about the positive vs. negative effects of COVID-19 vaccination that was experienced, or perhaps to a more general feeling of uncertainty. Although this kind of feeling should be common to all age groups, other studies could investigate more deeply how uncertain events impact individuals’ ambivalence focus in their cognitive and emotional processing.

Altogether, these issues coupled with our newly developed set of stimuli may have affected the general validity of our study. However, we believe that by expanding our understanding of mixed emotions via the development of different paradigms, we may unravel the different facets of emotional ambivalence and its relation to individual differences (age, cognitive level, and mood). We ultimately believe that a word-related approach may be promising in this regard, as it allows the integration of event perception and cognitive linguistic theories in the study of mixed emotions.

## Figures and Tables

**Table 1:** Demographics and control variables.

	Young		Young-Old		Old-Old		F	*p*	df	ηp^2^
	M	SD	M	SD	M	SD				
Education	13.958	1.805	14.666	2.807	13.583	1.665	1.566	0.216	2.69	0.043
Digit forward	8.458	2.206	7.916	1.348	7.500	1.474	1.876	0.161	2.69	0.052
Digit backward	8.125	1.676	7.791	1.503	7.750	1.594	0.400	0.672	2.69	0.011
PANAS +	31.458	6.021	31.791	4.791	33.708	4.657	1.312	0.275	2.69	0.036
PANAS −	22.583	3.562	21.166	5.411	23.958	5.473	1.951	0.149	2.69	0.053
Verbal fluency	11.291	2.793	10.271	2.115	9.867	2.258	2.235	0.114	2.69	0.061
GDS			9.208	2.828	9.250	2.027	0.003	0.953	1.46	0.000
BDI	5.292	2.679								
MMSE			28.167	1.049	28.042	0.999	0.180	0.674	1.46	0.003

**Table 2:** Age x type of valence. PW = positive words; NW = negative words; NeuW = neutral words; MPW = mixed-positive words; MNW = mixed-negative words.

		Mean	SD
Young	PW	0.657	0.112
	NW	0.729	0.132
	NeuW	0.553	0.062
	MPW	0.493	0.125
	MNW	0.516	0.112
Young-Old	PW	0.675	0.118
	NW	0.543	0.077
	NeuW	0.493	0.063
	MPW	0.714	0.119
	MNW	0.618	0.088
Old-Old	PW	0.512	0.083
	NW	0.481	0.102
	NeuW	0.372	0.075
	MPW	0.572	0.081
	MNW	0.591	0.084

## Data Availability

Our dataset is available on the OpenScience Framework project website at https://osf.io/cfqbr/.
